# Effect of Vestibular Stimulation Exercises on Level of Consciousness in Patients With Intracerebral Hemorrhage

**DOI:** 10.7759/cureus.111415

**Published:** 2026-06-24

**Authors:** Mukta T Killedar, Suraj B Kanase

**Affiliations:** 1 Department of Neurosciences Physiotherapy, Krishna College of Physiotherapy, Krishna Vishwa Vidyapeeth (Deemed to be University), Karad, IND

**Keywords:** four score, glasgow coma scale, intracerebral hemorrhage, level of consciousness, multisensory stimulation, neurorehabilitation, randomized controlled trial, vestibular stimulation

## Abstract

Introduction

Intracerebral hemorrhage (ICH) is frequently associated with impaired consciousness due to disruption of cortical, subcortical, and brainstem arousal networks. Reduced consciousness limits participation in rehabilitation and highlights the need for safe early interventions to facilitate responsiveness. Conventional multisensory stimulation provides visual, auditory, tactile, and olfactory input to support arousal. Graded vestibular stimulation may offer additional benefit through head movements, positional changes, and visual-vestibular activities; however, its role in patients with ICH remains insufficiently explored. This study aimed to evaluate the effectiveness of adding graded vestibular stimulation exercises to conventional multisensory stimulation for improving the level of consciousness (LOC) in patients with ICH.

Methods

This randomized controlled parallel-group trial was conducted in the neuro-ICU of Krishna Vishwa Vidyapeeth (Deemed to be University), Karad. Thirty-six patients with ICH were randomly allocated to an experimental group or a control group (n = 18 each). Both groups received conventional multisensory stimulation. The experimental group additionally received a graded vestibular stimulation exercise protocol, modified according to the patient’s LOC, including head and head-trunk movements, head-end inclination and declination positioning, rocking-based vestibular stimulation, wheelchair mobilization, gaze stabilization, eye movement exercises, and optokinetic visual stimulation. Interventions were administered once daily for 14 consecutive days; total treatment exposure was greater in the experimental group because vestibular exercises were provided as an add-on intervention. LOC was assessed by a blinded assessor using the Glasgow Coma Scale (GCS) and Full Outline of UnResponsiveness (FOUR) score at baseline (Day 1), Day 7, and Day 14. Statistical significance was set at p < 0.05.

Results

Both groups demonstrated significant improvement in level-of-consciousness scores from baseline to Day 14 (p < 0.001). Baseline GCS and FOUR scores did not differ significantly between groups. On Day 7, the experimental group demonstrated significantly higher GCS scores than the control group (9.2 ± 1.3 vs. 8.1 ± 1.2; p = 0.018) and significantly higher FOUR scores (11.0 ± 1.6 vs. 9.6 ± 1.5; p = 0.013). On Day 14, GCS scores remained significantly higher in the experimental group (11.3 ± 1.4 vs. 10.1 ± 1.3; p = 0.010), as did FOUR scores (13.4 ± 1.7 vs. 11.8 ± 1.6; p = 0.005). From Day 1 to Day 14, mean GCS and FOUR scores increased by 4.5 and 5.2 points, respectively, in the experimental group, compared with 3.4 and 3.7 points in the control group.

Conclusions

The addition of graded vestibular stimulation exercises to conventional multisensory stimulation was associated with significantly higher GCS and FOUR scores at Day 7 and Day 14 than conventional multisensory stimulation alone in patients with ICH. As total treatment exposure was greater in the experimental group, the findings reflect the effect of the add-on intervention rather than the isolated effect of vestibular stimulation alone.

## Introduction

Intracerebral hemorrhage (ICH) is a severe subtype of stroke, accounting for approximately 10-15% of all stroke cases, and is associated with disproportionately high mortality and long-term disability [1,2]. Despite advances in acute neurocritical care, outcomes remain poor, with many survivors experiencing persistent neurological deficits, reduced functional independence, and impaired quality of life [1,3]. The level of consciousness (LOC) is an important prognostic indicator in patients with ICH, as reduced consciousness may reflect extensive cerebral injury, mass effect, cerebral edema, raised intracranial pressure, or disruption of brainstem and thalamocortical arousal pathways [1-5]. Impaired consciousness also restricts active participation in rehabilitation and may adversely influence the course of neurological recovery.

Current management of ICH primarily focuses on physiological stabilization, prevention of secondary brain injury, blood pressure control, management of intracranial pressure, and neurosurgical intervention when indicated [2,3]. Although these measures are essential for survival and neurological stabilization, patients with reduced LOC often remain dependent on supportive care and are unable to actively engage in rehabilitation. Therefore, safe early rehabilitation approaches aimed at enhancing arousal and responsiveness may have an important supportive role in the management of patients with impaired consciousness following ICH.

Conventional multisensory stimulation is used in patients with impaired consciousness to provide structured visual, auditory, tactile, olfactory, and, when clinically safe, gustatory sensory inputs. These stimuli are intended to facilitate behavioral responsiveness and promote interaction with the environment. However, conventional multisensory stimulation may not specifically address vestibular and pruriceptive inputs associated with head movement, postural orientation, and verticalization. The addition of graded vestibular stimulation exercises may therefore provide a clinically relevant adjunctive approach in patients with impaired consciousness.

Vestibular stimulation represents a promising neurophysiological approach in this context. The vestibular system, comprising the semicircular canals and otolith organs, provides continuous information regarding head movement, spatial orientation, and gravitational forces. Vestibular afferents project to the vestibular nuclei in the brainstem and are connected with the reticular formation, cerebellum, thalamus, and cortical regions involved in sensory integration, attention, and motor control [5,6]. Through these connections, vestibular input may influence brainstem arousal networks and cortical responsiveness, providing a rationale for its use in patients with reduced consciousness.

Evidence from neuroscience suggests that peripheral sensory and vestibular inputs can modulate conscious experience and multisensory processing [7]. In patients with disorders of consciousness, vestibular-based stimulation has been associated with behavioral improvement in selected cases, supporting its potential role as an adjunctive neurorehabilitation intervention [8]. Furthermore, vestibular stimulation and positional changes may influence cerebral hemodynamic responses and sensorimotor integration, although the clinical relevance of these mechanisms in patients with ICH remains to be established [9,10].

Clinical research in patients with severe brain injury has also highlighted the potential role of early mobilization strategies, including head-up tilt and verticalization, in supporting arousal and neurological recovery [11-15]. These interventions provide graded postural and gravitational sensory input and may facilitate physiological adaptation and responsiveness during early rehabilitation. However, evidence regarding structured vestibular stimulation exercises specifically aimed at improving LOC in patients with ICH remains limited.

In the present study, the experimental intervention was designed as an additional graded vestibular stimulation exercise protocol administered along with conventional multisensory stimulation. The protocol was modified according to the participant’s LOC. In patients with lower levels of consciousness, such as coma or stupor, the vestibular intervention focused predominantly on passive head movements, coordinated head-and-trunk movement through log rolling, and head-end inclination and declination positioning. These activities were intended to provide controlled vestibular and postural sensory input while maintaining patient safety and physiological tolerance.

As the participant’s clinical responsiveness improved, the additional vestibular protocol progressed to more active-assisted and functional activities, including bedside sitting, sit-to-stand training, rocking-based vestibular stimulation, wheelchair mobilization, gaze stabilization exercises, eye movement exercises, and optokinetic visual stimulation. These activities were selected to provide progressively greater vestibular, postural, and visual-vestibular stimulation according to the participant’s tolerance and ability to participate. Conventional multisensory stimulation is used in patients with impaired consciousness to provide structured visual, auditory, tactile, and olfactory inputs. Gustatory stimulation may be introduced only at higher levels of responsiveness, after speech-language pathologist (SLP) clearance, and according to swallowing safety and patient tolerance.

A key feature of the intervention was its stage-based structure, which permitted modification of treatment intensity, complexity, and patient participation according to neurological status and physiological tolerance. Although some sensory stimulation components were repeated across levels of consciousness, their dosage and therapeutic emphasis were modified according to the participant’s responsiveness. Safety monitoring was incorporated throughout the intervention to minimize the risk of overstimulation, autonomic instability, or other adverse clinical responses.

Although vestibular stimulation, multisensory stimulation, and early mobilization strategies have been explored in neurological rehabilitation, evidence regarding the added effect of a graded vestibular stimulation exercise protocol alongside conventional multisensory stimulation in patients with ICH and impaired consciousness remains limited. Further investigation of such an approach is relevant because patients with reduced LOC require interventions that are safe, adaptable, and feasible within the neuro-ICU setting.

Therefore, the present randomized controlled trial aimed to evaluate the effectiveness of adding graded vestibular stimulation exercises to conventional multisensory stimulation for improving the LOC in patients with ICH. The control group received conventional multisensory stimulation alone, whereas the experimental group received conventional multisensory stimulation with additional graded vestibular stimulation exercises over a two-week intervention period. LOC was assessed using the Glasgow Coma Scale (GCS) and the Full Outline of UnResponsiveness (FOUR) score. It was hypothesized that participants receiving additional graded vestibular stimulation exercises would demonstrate greater improvement in consciousness scores than those receiving conventional multisensory stimulation alone.

## Materials and methods

The study received approval from the Institutional Ethics Committee of Krishna Vishwa Vidyapeeth (Deemed to be University), Karad (approval no. KVV/IEC/10/2025) prior to the commencement of the study. The study was conducted in accordance with ethical principles governing human research, with emphasis on participant safety, confidentiality, and adherence to institutional guidelines. As the participants had impaired consciousness, informed consent was obtained from legally authorized representatives or caregivers before enrollment.

Participants were recruited using a consecutive sampling technique from eligible patients admitted to the neuro-ICU during the study period. A total of 36 participants were enrolled and randomly allocated into two groups, with 18 participants assigned to each group. The sample size was determined based on feasibility considerations, availability of eligible participants, and the study duration; therefore, a formal a priori power analysis was not performed. Random sequence generation was carried out using a computer-based randomization method, and allocation concealment was ensured using sequentially numbered, opaque, sealed envelopes.

Patients were included if they had a confirmed diagnosis of spontaneous ICH based on neuroimaging findings and demonstrated an impaired LOC, defined as a GCS score of 12 or less [16] or a FOUR score of less than 16 [17]. Eligible participants were required to be hemodynamically stable, medically cleared for physiotherapy-based sensory and positional interventions, and aged between 55 and 65 years. The age range of 55-65 years was selected to reduce age-related variability in recovery potential, comorbidities, and rehabilitation outcomes, thereby improving sample homogeneity. Both male and female patients were included. Patients were excluded if they had known vestibular disorders, such as Ménière’s disease, vestibular neuritis, or labyrinthitis, that could interfere with vestibular responsiveness. Individuals with significant cerebral edema, evidence of uncontrolled intracranial hypertension, or unstable neurological status were excluded for safety reasons. Additional exclusion criteria included uncontrolled hypertension, active infection, cardiac arrhythmias, fractures, cervical spine injury, or musculoskeletal limitations restricting head or trunk movement. Patients receiving deep sedation, continuous sedative infusion, or neuromuscular blocking agents that could substantially interfere with consciousness assessment were excluded. Individuals with ear-related pathologies, such as otitis media, tympanic membrane perforation, or mastoid disease, were also excluded because these conditions could contraindicate vestibular stimulation. Patients with traumatic brain injury, traumatic ICH, subdural hematoma, epidural hematoma, traumatic subarachnoid hemorrhage, or other traumatic neurological conditions were also excluded from the study.

Participants were randomly allocated to either the experimental group or the control group. The intervention period consisted of 14 consecutive days, with treatment administered once daily according to group allocation. The intensity and progression of intervention were modified according to the participant’s LOC, physiological tolerance, and clinical responsiveness.

Both groups received conventional multisensory stimulation, consisting of visual stimulation, auditory stimulation using familiar voices and music, tactile stimulation, and olfactory stimulation. Gustatory stimulation was administered only to participants at the obtunded or lethargy stage, after clearance by an SLP, and according to swallowing safety and patient tolerance. Gustatory stimulation was not administered to participants at the coma or stupor stage.

The conventional multisensory stimulation protocol was informed by previously described sensory stimulation and disorders-of-consciousness rehabilitation approaches [18-20]. In addition to conventional multisensory stimulation, the experimental group received a graded vestibular stimulation exercise protocol. The vestibular and verticalization components were incorporated with reference to literature supporting vestibular stimulation, positional stimulation, and early mobilization in disorders of consciousness [8,11,21,22].

The additional graded vestibular stimulation exercise protocol included passive or active-assisted head movements, coordinated movement of the head and trunk through log rolling, head-end inclination and declination positioning, rocking-based vestibular stimulation, wheelchair mobilization with gradual changes in speed and direction, gaze stabilization exercises, eye movement exercises, and optokinetic visual stimulation. The intervention was modified according to the participant’s LOC. For participants at the coma or stupor stage, the protocol focused mainly on passive vestibular and sensory stimulation. In participants at the obtunded or lethargy stage, progressively more active-assisted and functional activities were included, such as bedside sitting, sit-to-stand training, wheelchair mobilization, visual tracking, gaze stabilization, and optokinetic visual stimulation.

Although certain conventional multisensory stimulation components were repeated across different levels of consciousness, their duration, complexity, level of patient participation, and therapeutic emphasis were modified according to clinical responsiveness and tolerance. Head-end inclination and declination positioning referred to the gradual elevation of the head end of the bed to the specified angle, followed by a return to the initial elevated position; it did not involve head-down positioning. Active-assisted exercises and functional activities, including bedside sitting, sit-to-stand training, wheelchair mobilization, gaze stabilization, and standing-based activities, were administered only when the participant demonstrated adequate medical stability, postural tolerance, and clinical responsiveness.

The control group received conventional multisensory stimulation alone and did not receive the additional graded vestibular stimulation exercise protocol. As vestibular stimulation exercises were added to conventional multisensory stimulation in the experimental group, the total intervention duration was longer in the experimental group than in the control group. Therefore, the present study evaluated the effectiveness of adding graded vestibular stimulation exercises to conventional multisensory stimulation, rather than the isolated effect of vestibular stimulation under time-matched treatment conditions.

The detailed conventional multisensory stimulation protocol administered to both groups and the additional graded vestibular stimulation exercise protocol administered to the experimental group, modified according to the participant’s LOC, are presented in Table 1.

**Table 1:** Conventional multisensory stimulation and additional graded vestibular stimulation exercise protocol according to the stage of consciousness LOC, level of consciousness; SLP, speech-language pathologist

Stage of consciousness	Group A: Experimental group (vestibular stimulation exercises + conventional multisensory stimulation)	Group B: Control group (conventional multisensory stimulation)
Coma	(1) Passive head movements: (a) Slow passive cervical flexion-extension movements (10 repetitions). (b) Slow passive cervical rotation movements from side to side (10 repetitions).	(1) Visual stimulation: (a) Ensure that the patient’s head is properly supported, usually at approximately 30° elevation. (b) The eyelids are gently opened while avoiding pressure on the eyeball. (c) Flashlight-based visual stimulation is administered by exposing each eye to intermittent bright light for one second on and one second off, repeated continuously for 10 seconds for each eye.
	(2) Passive movement of head and trunk together (log rolling) supine-to-side-lying turns: (a) The patient is gently turned from the supine position to alternate side-lying positions while maintaining coordinated movement of the head and trunk. (b) The activity is performed slowly and carefully for 10 repetitions.	(2) Auditory stimulation: (a) Auditory stimulation using familiar music and voice (two to three minutes).
	(3) Head-end inclination and declination positioning head-end inclination and declination positioning: (a) The head end of the bed is gradually elevated from 30° to 45° and returned to 30° in increments of 5°, according to patient tolerance. (b) The positioning activity is performed for 10 repetitions.	(3) Tactile stimulation: (a) Light tactile stimulation of the limbs and trunk (three to four minutes).
	(4) Visual stimulation: (a) Ensure that the patient’s head is properly supported, usually at approximately 30° elevation. (b) The eyelids are gently opened while avoiding pressure on the eyeball. (c) Flashlight-based visual stimulation is administered by exposing each eye to intermittent bright light for one second on and one second off, repeated continuously for 10 seconds for each eye.	(4) Olfactory and gustatory stimulation: (a) Olfactory stimulation using familiar pleasant odors (one minute).
	(5) Auditory stimulation: (a) Auditory stimulation using familiar music and voice (two to three minutes).	-
	(6) Tactile stimulation: (a) Light tactile stimulation of the limbs and trunk (three to four minutes).	
	(7) Olfactory and gustatory stimulation: (a) Olfactory stimulation using familiar pleasant odors and minimal safe taste stimuli if medically appropriate (one minute each).	
Stupor	(1) Passive head movements: (a) Passive cervical movements are performed initially at a slow speed and gradually progressed to moderate speed according to patient tolerance. (b) Passive cervical flexion-extension movements (10 repetitions). (c) Passive cervical rotation movements from side to side (10 repetitions).	(1) Visual stimulation: (a) Ensure that the patient’s head is properly supported, usually at approximately 30° elevation. (b) The eyelids are gently opened while avoiding pressure on the eyeball. (c) Flashlight-based visual stimulation is administered by exposing each eye to intermittent bright light for one second on and one second off, repeated continuously for 10 seconds for each eye.
	(2) Passive movement of head and trunk together (log rolling) supine-to-side-lying turns: (a) The patient is gently turned from the supine position to alternate side-lying positions while maintaining coordinated movement of the head and trunk. (b) The activity is performed slowly and carefully for 10 repetitions.	(2) Auditory stimulation (a) Auditory stimulation using familiar music and voice (three to five minutes).
	(3) Head-end inclination and declination positioning: (a) The head end of the bed is gradually elevated from 45° to 50° and returned to 45° in increments of 5°, according to patient tolerance. (b) The positioning activity is performed for 10 repetitions.	(3) Tactile stimulation: (a) Fingertip pressure and stimulation using textured surfaces (four to six minutes).
	(4) Visual stimulation: (a) Ensure that the patient’s head is properly supported, usually at approximately 30° elevation. (b) The eyelids are gently opened while avoiding pressure on the eyeball. (c) Flashlight-based visual stimulation is administered by exposing each eye to intermittent bright light for one second on and one second off, repeated continuously for 10 seconds for each eye.	(4) Olfactory stimulation: (a) Olfactory stimulation using familiar pleasant odors (one to two minutes).
	(5) Auditory stimulation: (a) Auditory stimulation using familiar music and voice (three to five minutes).	-
	(6) Tactile stimulation: (a) Fingertip pressure and stimulation using textured surfaces (four to six minutes).	
	(7) Olfactory stimulation: (a) Olfactory stimulation using familiar pleasant odors (one minute).	
Obtunded	(1) Active-assisted head movements: (a) Active-assisted cervical movements are performed initially at a slow speed and gradually progressed to moderate speed according to patient tolerance. (b) Active-assisted cervical flexion-extension movements (10 repetitions). (c) Active-assisted cervical rotation movements from side to side (10 repetitions).	(1) Visual stimulation: (a) Ensure that the patient’s head is properly supported, usually at approximately 30° elevation. (b) Utilize brightly colored objects, a mirror, or pictures of various shapes and sizes. (c) Encourage the patient to visually fixate on and track the presented objects.
	(2) Active-assisted movement of head and trunk together (log rolling) supine-to-side-lying turns: (a) The patient is gently assisted from the supine position to alternate side-lying positions while maintaining coordinated movement of the head and trunk. (b) The activity is performed for 10 repetitions.	(2) Auditory stimulation: (a) Auditory stimulation using familiar music and voice (five minutes).
	(3) Head-end inclination and declination positioning: (a) The head end of the bed is gradually elevated from 45° to 60° and returned to 45° in increments of 5°, according to patient tolerance. (b) The positioning activity is performed for 15 repetitions.	(3) Tactile stimulation: (a) Tactile stimulation using textured surfaces, temperature stimuli, and gentle hand compression (five minutes).
	(4) Bedside sitting: (a) Assisted supported bedside sitting is performed for five to 10 minutes according to patient tolerance.	(4) Olfactory and gustatory stimulation: (a) Olfactory and gustatory stimulation using familiar odors and safe taste stimuli following SLP clearance (two to three minutes each).
	(5) Sit-to-stand training: (a) Assisted sit-to-stand activity is performed for five repetitions according to patient tolerance.	-
	(6) Passive rocking movement: (a) Vestibular rocking stimulation is administered using a rocking chair for five to 10 minutes according to patient tolerance.	
	(7) Wheelchair mobilization: (a) Wheelchair mobilization is performed with gradual changes in speed and direction of movement for five minutes according to patient tolerance.	
	(8) Visual stimulation: (a) Ensure that the patient’s head is properly supported, usually at approximately 30° elevation. (b) Utilize brightly colored objects, a mirror, or pictures of various shapes and sizes. (c) Encourage the patient to visually fixate on and track the presented objects.	
	(9) Gaze stabilization exercises head turns (side-to-side): (a) Hold the visual target steady while the patient slowly turns the head from side to side, maintaining visual fixation on the target throughout the movement for 10 repetitions (head nods (up-and-down)). (b) Hold the visual target steady while the patient slowly moves the head up and down, maintaining visual fixation on the target throughout the movement for 10 repetitions.	
	(10) Eye movement exercises: (a) Vertical eye movements (up and down). (b) Horizontal eye movements (side to side). (c) Near-far visual focusing exercises by tracking a finger moved gradually from approximately 3 feet to 1 foot away from the face.	
	(11) Optokinetic visual stimulation: (a) Optokinetic visual stimulation is administered with the patient in a supported supine or semi-Fowler’s position while ensuring proper head alignment and stability. (b) A high-contrast moving striped stimulus is positioned approximately 30-50 cm from the eyes and moved horizontally in both directions for 30-60 seconds each. (c) The patient is encouraged to visually track the stimulus while monitoring eye movements and alertness. (d) The activity is performed for five minutes once daily with continuous monitoring of vital signs and patient tolerance with the intended aim of promoting arousal while continuously monitoring vital signs and patient tolerance.	
	(12) Auditory stimulation: (a) Auditory stimulation using familiar music and voice (five minutes).	
	(13) Tactile stimulation: (a) Tactile stimulation using textured surfaces, temperature stimuli, and gentle hand compression (five minutes).	
	(14) Olfactory and gustatory stimulation: (a) Olfactory and gustatory stimulation using familiar odors and safe taste stimuli following SLP clearance (two to three minutes each).	
Lethargy	(1) Active-assisted head movements: (a) Active-assisted cervical movements are performed initially at a slow speed and gradually progressed to moderate speed according to patient tolerance. (b) Active-assisted cervical flexion-extension movements (10 repetitions). (c) Active-assisted cervical rotation movements from side to side (10 repetitions).	(1) Visual stimulation: (a) Ensure that the patient’s head is properly supported, usually at approximately 30° elevation. (b) Utilize brightly colored objects, a mirror, or pictures of various shapes and sizes. (c) Encourage the patient to visually fixate on and track the presented objects.
	(2) Active-assisted movement of head and trunk together (log rolling) supine-to-side-lying turns: (a) The patient is gently assisted from the supine position (lying on the back) to alternate side-lying positions while maintaining coordinated movement of the head and trunk. (b) The activity is performed for 10 repetitions.	(2) Auditory stimulation: (a) Auditory stimulation using familiar music and voice (five minutes).
	(3) Bedside sitting: (a) Assisted supported bedside sitting is performed for 10 minutes according to patient tolerance.	(3) Tactile stimulation: (a) Tactile stimulation using object manipulation activities with varied textures, shapes, and sizes to promote sensory awareness and purposeful upper limb activity (five to seven minutes).
	(4) Sit-to-stand training: (a) Assisted sit-to-stand activity is performed for 10 repetitions according to patient tolerance.	(4) Olfactory and gustatory stimulation: (a) Olfactory and gustatory stimulation using familiar odors and safe taste stimuli following SLP clearance (three to four minutes).
	(5) Passive rocking movement: (a) Vestibular rocking stimulation is administered using a rocking chair for 10 minutes according to patient tolerance.	-
	(6) Wheelchair mobilization: (a) Wheelchair mobilization is performed with gradual changes in speed and direction of movement for five minutes according to patient tolerance.	
	(7) Visual stimulation: (a) Ensure that the patient’s head is properly supported, usually at approximately 30° elevation. (b) Utilize brightly colored objects, a mirror, or pictures of various shapes and sizes. (c) Encourage the patient to visually fixate on and track the presented objects.	
	(8) Advanced gaze stabilization exercises head and target movements in opposite directions: (a) The patient is positioned in sitting or standing with a visual target placed at eye level approximately an arm’s length away. (b) The patient is instructed to maintain visual fixation on the target while keeping the image clear and stable throughout the activity. (c) Exercises are initiated with horizontal movements and gradually progressed to vertical movements according to patient tolerance (head turns (side-to-side)). (d) The patient slowly turns the head from side to side while simultaneously moving the target in the opposite direction, maintaining visual fixation throughout the movement for 10 repetitions (head nods (up-and-down)). (e) The patient slowly moves the head up and down while simultaneously moving the target in the opposite direction, maintaining visual fixation throughout the movement for 10 repetitions.	
	(9) Optokinetic visual stimulation: (a) Optokinetic visual stimulation is administered with the patient in a supported supine or semi-Fowler’s position while ensuring proper head alignment and stability. (b) A high-contrast moving striped stimulus is positioned approximately 30-50 cm from the eyes and moved horizontally in both directions for 30-60 seconds each. (c) The patient is encouraged to visually track the stimulus while monitoring eye movements and alertness. (d) The activity is performed for five minutes once daily with continuous monitoring of vital signs and patient tolerance to promote arousal and improve LOC.	
	(10) Auditory stimulation: (a) Auditory stimulation using familiar music and voice (five minutes).	
	(11) Tactile stimulation: (a) Tactile stimulation using object manipulation activities with varied textures, shapes, and sizes to promote sensory awareness and purposeful upper limb activity (five to seven minutes).	
	(12) Olfactory and gustatory stimulation: (a) Olfactory and gustatory stimulation using familiar odors and safe taste stimuli following SLP clearance (three to four minutes).	

During intervention sessions, participants were closely monitored for physiological stability, including heart rate, blood pressure, respiratory status, oxygen saturation (SpO₂), and neurological responsiveness. Intervention intensity, duration, or progression was modified or temporarily discontinued in the event of hemodynamic instability, excessive autonomic response, respiratory compromise, reduced tolerance, or clinical deterioration. Appropriate precautions were implemented to minimize the risk of overstimulation, autonomic instability, aspiration during gustatory stimulation, or other adverse clinical events.

The primary outcome was the LOC, assessed using the GCS [16] and the FOUR score [17]. The GCS evaluates eye opening, verbal response, and motor response, with scores ranging from 3 to 15, where lower scores indicate greater impairment of consciousness. The FOUR score provides a broader neurological assessment by evaluating eye response, motor response, brainstem reflexes, and respiratory pattern, with total scores ranging from 0 to 16, where lower scores reflect more severe neurological impairment and reduced responsiveness, making it particularly applicable in critically ill neurological patients. Outcome assessments were conducted at three predefined time points: baseline assessment on Day 1, mid-intervention assessment on Day 7, and post-intervention assessment on Day 14. To minimize measurement bias, all evaluations were performed by a blinded assessor who was not involved in participant allocation or intervention delivery.

Statistical analysis was performed using IBM SPSS Statistics for Windows, version 31.0 (released 2025; IBM Corp., Armonk, NY, USA). Descriptive statistics were used to summarize demographic and clinical characteristics. Continuous variables, including age, GCS scores, and FOUR scores, were expressed as mean ± SD, whereas categorical variables, such as gender, were presented as frequency and percentage. The Shapiro-Wilk test was used to assess the normality of the data distribution. Between-group comparisons of GCS and FOUR scores at each assessment time point (Day 1, Day 7, and Day 14) were performed using independent-samples t-tests. Within-group comparisons of GCS and FOUR scores from baseline (Day 1) to post-intervention assessment (Day 14) were performed using paired-samples t-tests. Cohen’s d was calculated to estimate the magnitude of between-group and within-group differences. A p-value of <0.05 was considered statistically significant.

All interventions were noninvasive and administered under continuous clinical supervision within the neuro-ICU setting.

## Results

A total of 36 participants were included in the study and randomly allocated to the experimental and control groups, with 18 participants in each group. Baseline demographic characteristics were comparable between the groups. The mean age of participants in the experimental group was 60.2 ± 3.1 years, while that of participants in the control group was 59.8 ± 3.4 years, with no statistically significant difference between groups (t = 0.36, p = 0.72). Gender distribution was identical in both groups, with nine males (50%) and nine females (50%) in each group, as shown in Table 2.

**Table 2:** Baseline demographic characteristics of participants

Variable	Experimental group (n = 18)	Control group (n = 18)	Test value	p-value
Age (years), mean ± SD	60.2 ± 3.1	59.8 ± 3.4	t = 0.36	0.72
Gender, n (%)			χ² = 0.00	1
Male	9 (50%)	9 (50%)	-	-
Female	9 (50%)	9 (50%)	-	-

At baseline (Day 1), no statistically significant difference was observed in GCS scores between the experimental group (6.8 ± 1.2) and the control group (6.7 ± 1.3) (t = 0.23, p = 0.82; Cohen’s d = 0.08). On Day 7, the experimental group demonstrated significantly higher GCS scores than the control group (9.2 ± 1.3 vs. 8.1 ± 1.2; t = 2.48, p = 0.018; Cohen’s d = 0.88). On Day 14, the experimental group continued to demonstrate significantly higher GCS scores than the control group (11.3 ± 1.4 vs. 10.1 ± 1.3; t = 2.72, p = 0.010; Cohen’s d = 0.89) as shown in Table 3.

**Table 3:** Between-group comparison of GCS scores across assessment time points GCS, Glasgow Coma Scale

Time point	Experimental (mean ± SD)	Control (mean ± SD)	Mean difference	t-value	p-value	Cohen’s d	Effect size
Day 1	6.8 ± 1.2	6.7 ± 1.3	0.1	0.23	0.82	0.08	Negligible
Day 7	9.2 ± 1.3	8.1 ± 1.2	1.1	2.48	0.018	0.88	Large
Day 14	11.3 ± 1.4	10.1 ± 1.3	1.2	2.72	0.01	0.89	Large

A similar pattern was observed for FOUR scores. At baseline (Day 1), the FOUR scores were comparable between the experimental group (8.2 ± 1.5) and the control group (8.1 ± 1.4), with no statistically significant difference between groups (t = 0.15, p = 0.88; Cohen’s d = 0.07). On Day 7, the experimental group demonstrated significantly higher FOUR scores than the control group (11.0 ± 1.6 vs. 9.6 ± 1.5; t = 2.65, p = 0.013; Cohen’s d = 0.90). On Day 14, the experimental group continued to demonstrate significantly higher FOUR scores than the control group (13.4 ± 1.7 vs. 11.8 ± 1.6; t = 3.05, p = 0.005; Cohen’s d = 0.97) as shown in Table 4.

**Table 4:** Between-group comparison of FOUR scores across assessment time points FOUR, Full Outline of UnResponsiveness

Time point	Experimental (mean ± SD)	Control (mean ± SD)	Mean difference	t-value	p-value	Cohen’s d	Effect size
Day 1	8.2 ± 1.5	8.1 ± 1.4	0.1	0.15	0.88	0.07	Negligible
Day 7	11.0 ± 1.6	9.6 ± 1.5	1.4	2.65	0.013	0.9	Large
Day 14	13.4 ± 1.7	11.8 ± 1.6	1.6	3.05	0.005	0.97	Large

Within-group analysis demonstrated significant improvement in GCS scores from baseline to post-intervention in both groups. In the experimental group, mean GCS scores increased from 6.8 ± 1.2 on Day 1 to 11.3 ± 1.4 on Day 14, corresponding to a mean change of 4.5 points (t = 6.10, p < 0.001; Cohen’s d = 1.44). In the control group, mean GCS scores increased from 6.7 ± 1.3 on Day 1 to 10.1 ± 1.3 on Day 14, corresponding to a mean change of 3.4 points (t = 5.20, p < 0.001; Cohen’s d = 1.23) as shown in Table 5.

**Table 5:** Within-group comparison of GCS scores GCS, Glasgow Coma Scale

Group	Day 1	Day 14	Mean change	t-value	p-value	Cohen’s d	Effect size
Experimental	6.8 ± 1.2	11.3 ± 1.4	4.5	6.1	<0.001	1.44	Very large
Control	6.7 ± 1.3	10.1 ± 1.3	3.4	5.2	<0.001	1.23	Large

Within-group analysis of FOUR scores also demonstrated significant improvement from baseline to post-intervention in both groups. In the experimental group, mean FOUR scores increased from 8.2 ± 1.5 on Day 1 to 13.4 ± 1.7 on Day 14, corresponding to a mean change of 5.2 points (t = 7.10, p < 0.001; Cohen’s d = 1.67). In the control group, mean FOUR scores increased from 8.1 ± 1.4 on Day 1 to 11.8 ± 1.6 on Day 14, corresponding to a mean change of 3.7 points (t = 5.80, p < 0.001; Cohen’s d = 1.37) as shown in Table 6.

**Table 6:** Within-group comparison of FOUR scores FOUR, Full Outline of UnResponsiveness

Group	Day 1	Day 14	Mean change	t-value	p-value	Cohen’s d	Effect size
Experimental	8.2 ± 1.5	13.4 ± 1.7	5.2	7.1	<0.001	1.67	Very large
Control	8.1 ± 1.4	11.8 ± 1.6	3.7	5.8	<0.001	1.37	Very large

Overall, both groups demonstrated significant improvement in GCS and FOUR scores from baseline to post-intervention. At Day 7 and Day 14, the experimental group, which received additional graded vestibular stimulation exercises along with conventional multisensory stimulation, demonstrated significantly higher GCS and FOUR scores than the control group, which received conventional multisensory stimulation alone. These findings indicate that the add-on graded vestibular stimulation protocol was associated with more favorable level-of-consciousness scores during the intervention period. As the experimental group received greater total treatment exposure, the findings should be interpreted as reflecting the effect of the add-on intervention rather than the isolated effect of vestibular stimulation alone.

## Discussion

The present study evaluated the effectiveness of adding a graded vestibular stimulation exercise protocol to conventional multisensory stimulation for improving the LOC in patients with ICH. Both the experimental and control groups demonstrated significant improvement in GCS and FOUR scores from baseline to post-intervention. At Day 7 and Day 14, the experimental group, which received additional graded vestibular stimulation exercises along with conventional multisensory stimulation, demonstrated significantly higher GCS and FOUR scores than the control group, which received conventional multisensory stimulation alone. These findings suggest that the add-on graded vestibular stimulation protocol may be associated with more favorable level-of-consciousness scores during the intervention period.

ICH is associated with substantial neurological impairment resulting from primary tissue injury and secondary pathological processes, including cerebral edema, raised intracranial pressure, mass effect, and disruption of neural networks involved in arousal and awareness. Witsch et al. [1] and Magid-Bernstein et al. [2] described the complex pathophysiological mechanisms contributing to neurological deterioration following ICH. Schrag and Kirshner [3] further emphasized the clinical importance of impaired consciousness in determining prognosis and limiting participation in rehabilitation. Although acute medical and surgical management remains essential for stabilization and survival, rehabilitation strategies specifically directed toward improving arousal and responsiveness in patients with reduced consciousness remain limited.

Conventional multisensory stimulation provides structured visual, auditory, tactile, and olfactory input to facilitate responsiveness and interaction with the environment. In the present study, conventional multisensory stimulation was administered to both groups, which may partly explain the significant improvement observed within the control group from baseline to post-intervention. The experimental group additionally received graded vestibular stimulation exercises. Therefore, the present study examined vestibular stimulation as an adjunct to conventional multisensory stimulation rather than as an isolated intervention.

From a neurophysiological perspective, vestibular stimulation may be relevant in patients with impaired consciousness because vestibular afferents project to brainstem vestibular nuclei and are functionally connected with the reticular formation, cerebellum, thalamus, and cortical regions involved in sensory integration, attention, and motor control. The reticular formation contributes to the ascending reticular activating system, which plays an important role in maintaining arousal and wakefulness. Bottini et al. [7] reported that peripheral sensory stimulation can modulate conscious experience through distributed neural systems. Serrador et al. [9] also described the effects of vestibular stimulation on cerebral blood flow regulation. Together, these findings provide a plausible physiological rationale for incorporating vestibular input into rehabilitation for patients with impaired consciousness. However, as the present study did not directly assess cerebral perfusion or neural activation, these mechanisms remain theoretical explanations for the observed clinical findings.

The graded vestibular stimulation exercise protocol used in this study included passive and active-assisted head movements, coordinated head-and-trunk movements through log rolling, head-end inclination and declination positioning, rocking-based vestibular stimulation, wheelchair mobilization, gaze stabilization exercises, eye movement exercises, and optokinetic visual stimulation. The protocol was modified according to the participant’s LOC and clinical tolerance. Participants at the coma and stupor stages received predominantly passive vestibular and positional stimulation, whereas participants at the obtunded and lethargy stages received progressively more active-assisted and functional components. This stage-based approach allowed the intervention to be adapted to clinical responsiveness, postural tolerance, and physiological stability.

At baseline, GCS and FOUR scores were comparable between the groups. At Day 7 and Day 14, the experimental group demonstrated significantly higher GCS scores than the control group. This suggests that participants receiving additional graded vestibular stimulation exercises alongside conventional multisensory stimulation had more favorable clinical consciousness scores during the intervention period. Similarly, FOUR scores were significantly higher in the experimental group at Day 7 and Day 14. Because the FOUR score includes eye response, motor response, brainstem reflexes, and respiratory pattern, it provides clinically useful information regarding neurological responsiveness in critically ill patients. Nevertheless, the higher FOUR scores should not be interpreted as direct evidence of enhanced brainstem function because the study did not include objective neurophysiological measures.

The findings of the present study are consistent with previous literature examining vestibular and positional approaches in patients with disorders of consciousness or severe neurological impairment. Vanzan et al. [8] reported behavioral improvement following caloric vestibular stimulation in individuals with a minimally conscious state. Although caloric vestibular stimulation differs from the exercise-based protocol used in the present study, their findings support the potential relevance of vestibular input in disorders of consciousness. Studies investigating head-up tilt and verticalization have also suggested possible benefits in patients with impaired consciousness [11,12,15,19,20]. Krewer et al. [22] and Frazzitta et al. [23] highlighted the clinical relevance of verticalization and mobilization-related interventions in patients with severe brain injury. In the present study, head-end positioning, bedside sitting, sit-to-stand activities, and wheelchair mobilization were incorporated within a broader graded vestibular stimulation framework.

Visual-vestibular interaction may also have contributed to the clinical findings in participants who were able to respond to more active tasks. The experimental protocol incorporated gaze stabilization exercises, eye movement exercises, and optokinetic visual stimulation at higher levels of consciousness. Cornell et al. [24] described the influence of vestibular-related stimulation on oculomotor responses through vestibulo-ocular pathways. These components may support visual attention, gaze control, and sensory integration when patients demonstrate sufficient responsiveness to participate. However, as multiple vestibular components were delivered together, the present study cannot determine which specific intervention component contributed most to the observed scores.

Laureys et al. [19] and Laureys et al. [20] described recovery of consciousness as involving distributed cortical and subcortical networks responsible for arousal, awareness, and behavioral responsiveness. The present protocol was clinically designed to provide progressively graded sensory input that included vestibular, postural, and visual-vestibular components, in addition to the conventional multisensory stimulation administered to both groups. This approach may be relevant in patients with reduced consciousness because it permits gradual advancement from passive input to more active-assisted stimulation as clinical responsiveness improves.

Both groups demonstrated significant improvement in GCS and FOUR scores from baseline to post-intervention. Improvement in the control group may reflect the effects of conventional multisensory stimulation, ongoing medical management, routine clinical care, and natural neurological recovery during the study period. The significantly higher GCS and FOUR scores observed in the experimental group at Day 7 and Day 14 suggest a possible additional benefit of the graded vestibular stimulation exercise protocol when added to conventional multisensory stimulation. However, because the experimental group received additional intervention components and greater total treatment exposure, the findings should be interpreted as reflecting the effect of the add-on graded vestibular stimulation program rather than establishing the isolated effect of vestibular stimulation alone.

Several limitations should be acknowledged. First, the sample size was relatively small, and no formal a priori power analysis was performed; therefore, the generalizability and statistical precision of the findings may be limited. Second, the intervention period was restricted to 14 days, and no follow-up assessment was conducted after completion of treatment. Consequently, the long-term sustainability of changes in GCS and FOUR scores cannot be determined. Third, the experimental group received additional graded vestibular stimulation exercises in addition to conventional multisensory stimulation, resulting in greater total treatment exposure than the control group. Therefore, the observed between-group differences may have been influenced by both the content of the vestibular stimulation protocol and the additional duration of therapy. As the treatment duration was not time-matched between groups, the potential contribution of increased treatment exposure cannot be excluded. Fourth, the intervention consisted of multiple graded vestibular components, preventing identification of the individual contribution of head movements, positional changes, rocking, verticalization, gaze stabilization, optokinetic stimulation, or mobility-based activities. Fifth, the study did not include objective neurophysiological or neuroimaging measures, such as electroencephalography, functional neuroimaging, or cerebral perfusion assessment, to verify the proposed mechanisms underlying the clinical findings. Sixth, detailed information regarding ICH location (e.g., supratentorial, infratentorial, brainstem, or cerebellar involvement) and hematoma size was not collected as part of the study protocol and therefore could not be analyzed. These factors may influence neurological severity, recovery trajectories, and responsiveness to rehabilitation interventions.

Future studies should include larger sample sizes, formal sample-size calculation, longer follow-up durations, and time-matched comparison groups to determine the specific effect of vestibular stimulation independent of additional treatment exposure. Ensuring equivalent treatment duration between study groups would help reduce potential confounding related to therapy dosage and allow a more accurate evaluation of the specific contribution of vestibular stimulation to the recovery of consciousness. Further research should also consider incorporating objective neurophysiological assessments to examine the relationship between vestibular stimulation and arousal-related neural activity in patients with impaired consciousness following ICH. Collection and analysis of hemorrhage location and hematoma size should also be considered in future studies to better characterize the study population and explore their potential influence on treatment outcomes. Studies comparing individual vestibular components or different dosage schedules may also help identify the most feasible and clinically useful intervention approach for neuro-ICU rehabilitation.

## Conclusions

The present study suggests that adding a graded vestibular stimulation exercise protocol to conventional multisensory stimulation may be clinically beneficial for patients with impaired consciousness following ICH. Participants who received additional graded vestibular stimulation exercises demonstrated significantly higher GCS and FOUR scores at Day 7 and Day 14 than those who received conventional multisensory stimulation alone. These findings suggest that graded vestibular stimulation may have potential as an adjunctive neurorehabilitation approach in the neuro-ICU setting; however, the specific contribution of vestibular stimulation independent of treatment duration cannot be definitively established.

However, the results should be interpreted cautiously because the experimental group received greater total treatment exposure than the control group; therefore, the findings reflect the effect of the add-on intervention rather than the isolated effect of vestibular stimulation alone. In addition, the limited sample size, short intervention period, and absence of objective neurophysiological measures restrict conclusions regarding long-term effectiveness and underlying mechanisms. Further randomized controlled studies with larger samples, time-matched comparison groups, longer follow-up periods, and objective measures such as electroencephalography or neuroimaging are recommended to confirm these findings.
